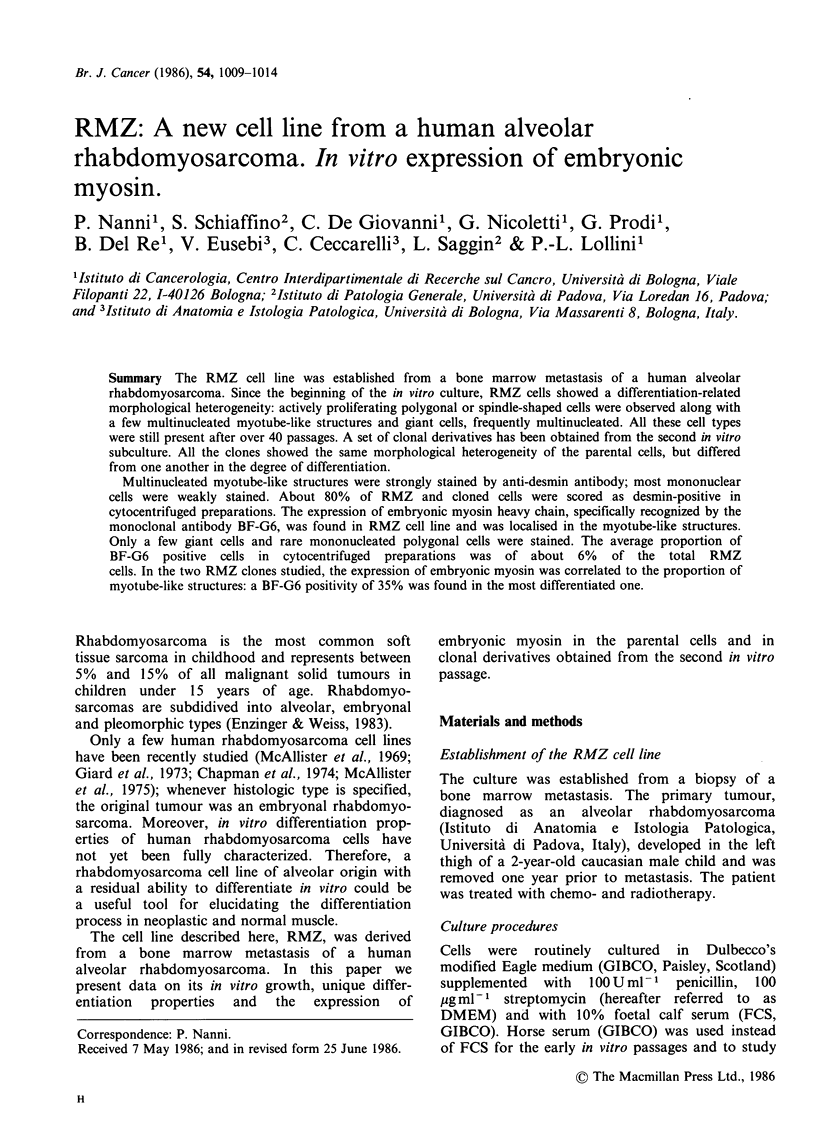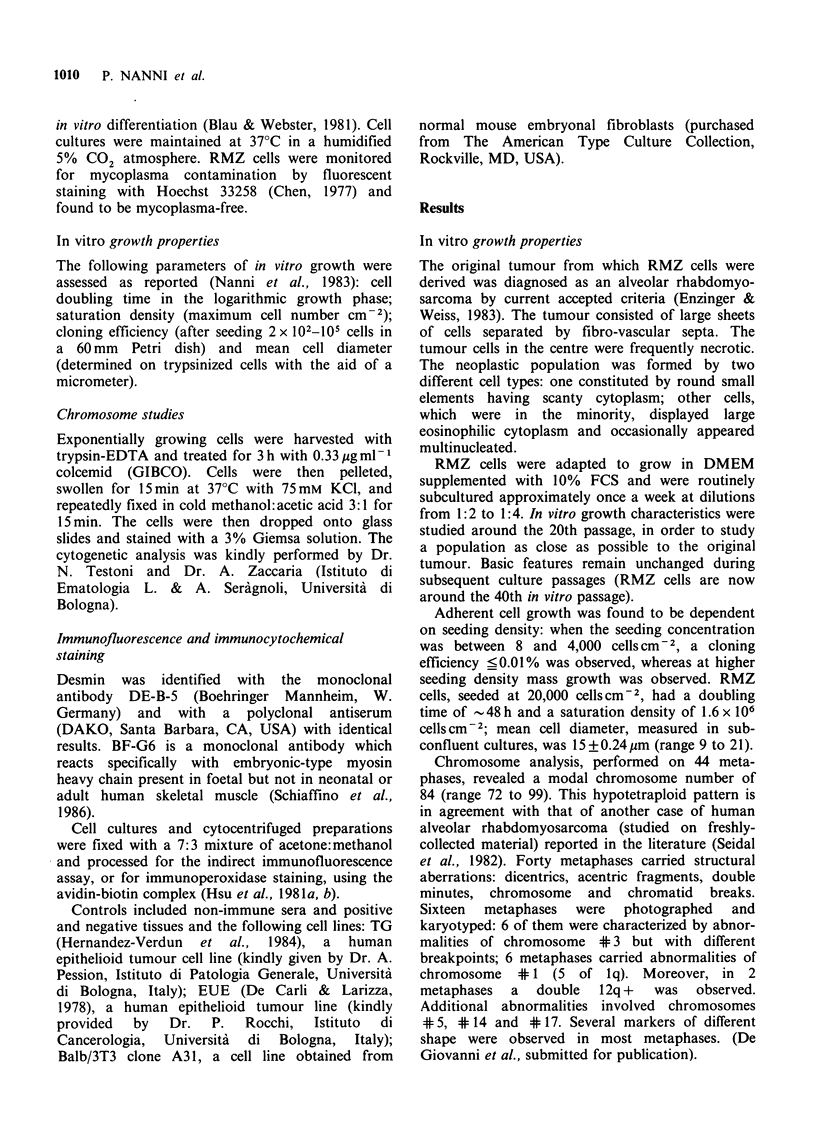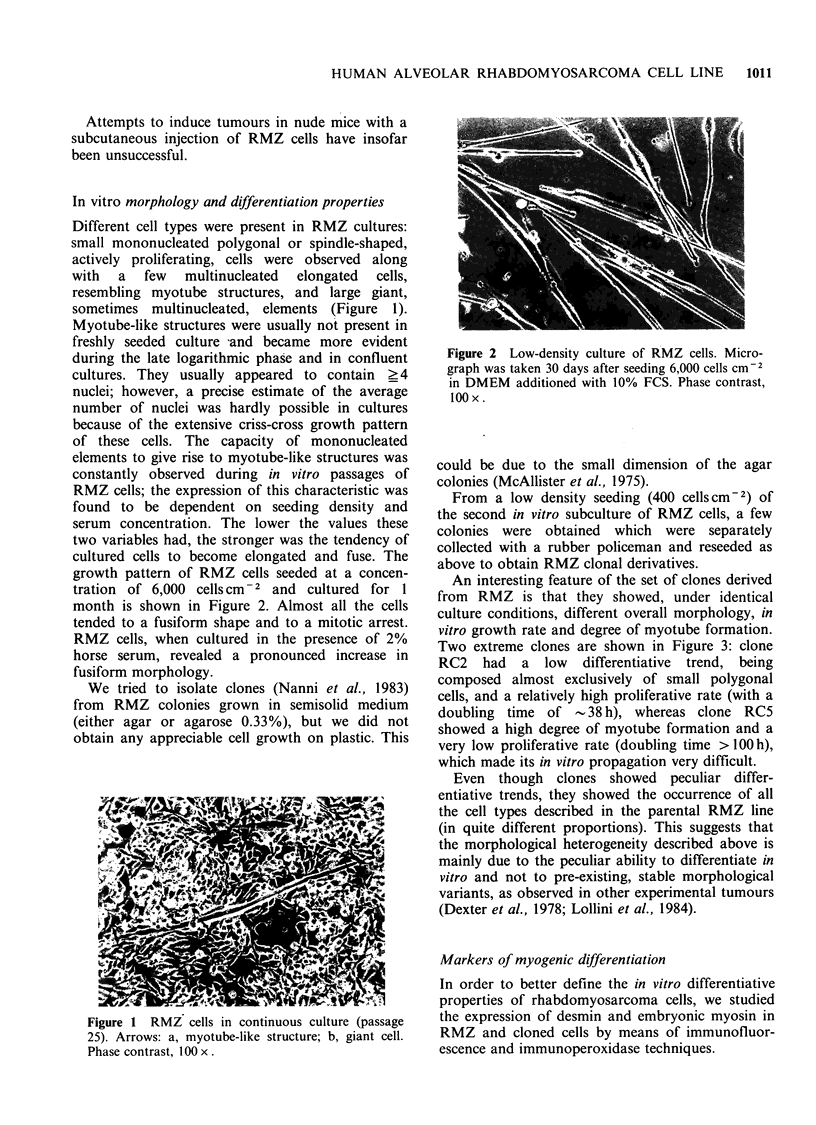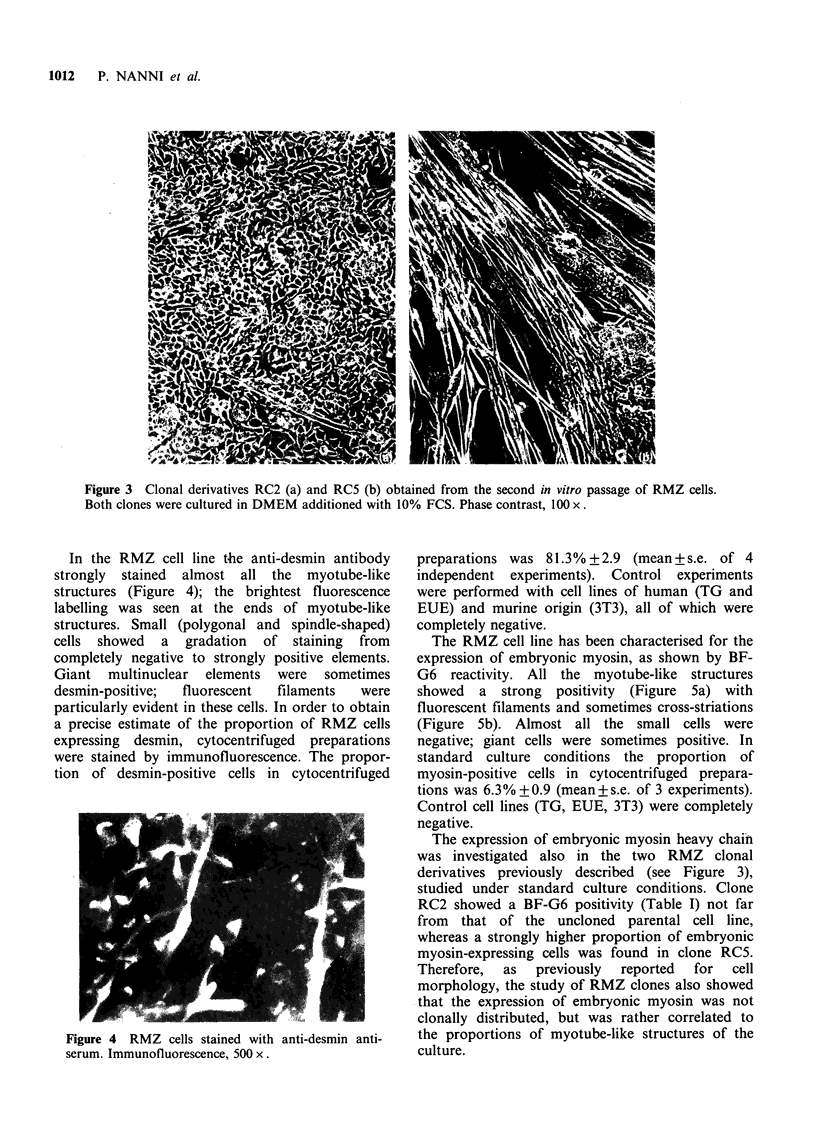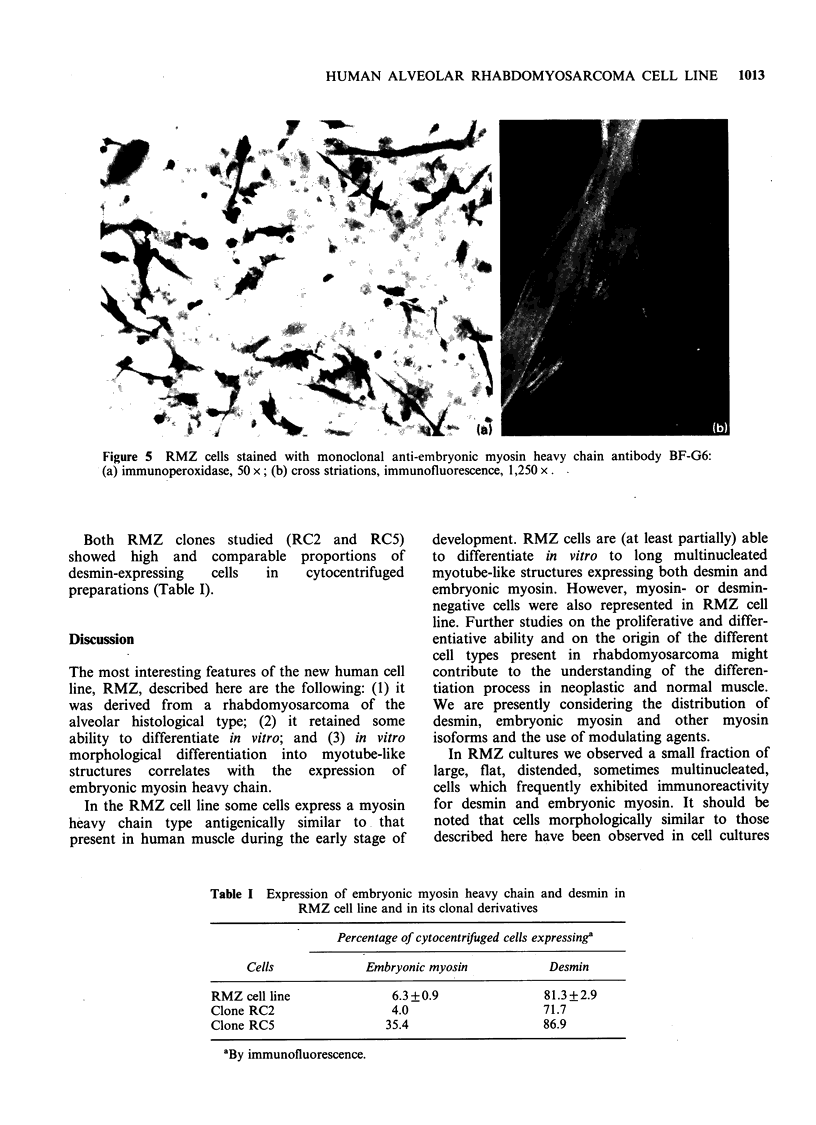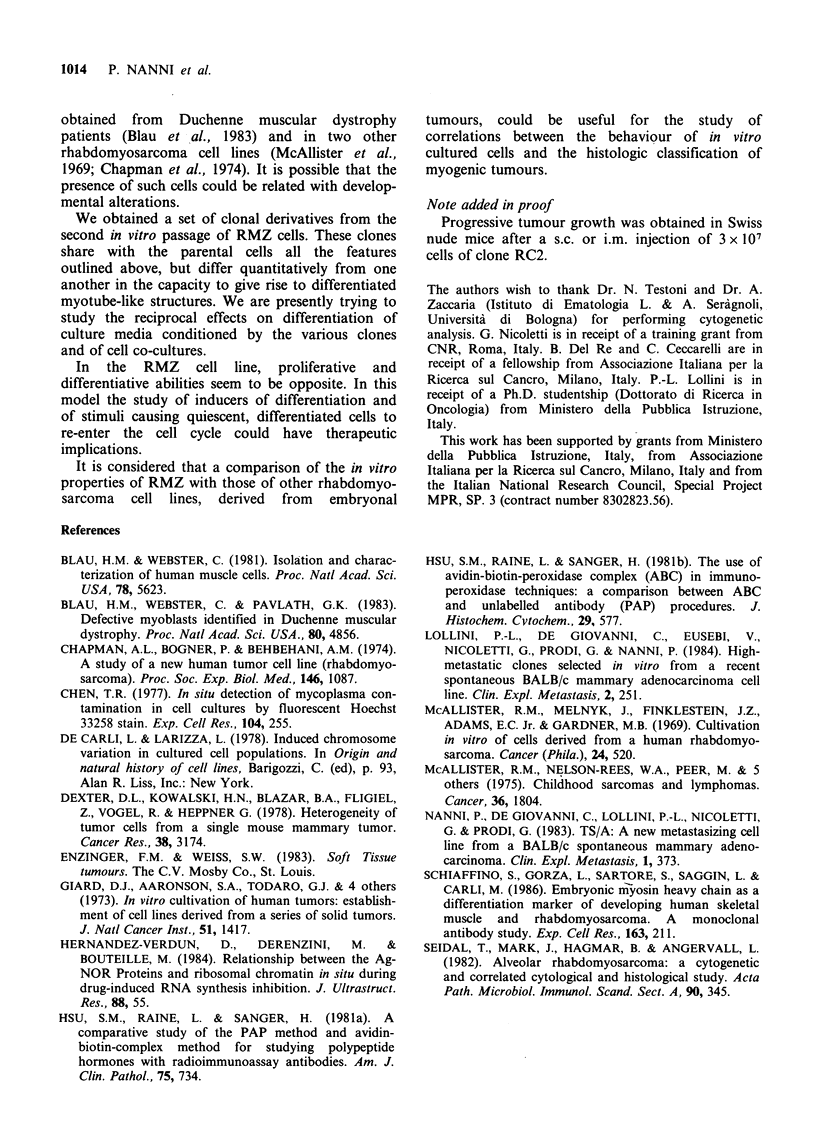# RMZ: a new cell line from a human alveolar rhabdomyosarcoma. In vitro expression of embryonic myosin.

**DOI:** 10.1038/bjc.1986.273

**Published:** 1986-12

**Authors:** P. Nanni, S. Schiaffino, C. De Giovanni, G. Nicoletti, G. Prodi, B. Del Re, V. Eusebi, C. Ceccarelli, L. Saggin, P. L. Lollini

## Abstract

**Images:**


Br. J. Cancer (1986), 54, 1009–1014

### RMZ: A new cell line from a human alveolar rhabdomyosarcoma. *In vitro* expression of embryonic myosin.

P. Nanni[1], S. Schiaffino[2], C. De Giovanni[1], G. Nicoletti[1], G. Prodi[1], B. Del Re[1], V. Eusebi[3], C. Ceccarelli[3], L. Saggin[2] & P.-L. Lollini[1]

[1]*Istituto di Cancerologia, Centro Interdipartimentale di Recerche sul Cancro, Università di Bologna, Viale Filopanti 22, I-40126 Bologna;* [2]*Istituto di Patologia Generale, Università di Padova, Via Loredan 16, Padova; and* [3]*Istituto di Anatomia e Istologia Patologica, Università di Bologna, Via Massarenti 8, Bologna, Italy.*

**Summary** The RMZ cell line was established from a bone marrow metastasis of a human alveolar rhabdomyosarcoma. Since the beginning of the *in vitro* culture, RMZ cells showed a differentiation-related morphological heterogeneity: actively proliferating polygonal or spindle-shaped cells were observed along with a few multinucleated myotube-like structures and giant cells, frequently multinucleated. All these cell types were still present after over 40 passages. A set of clonal derivatives has been obtained from the second *in vitro* subculture. All the clones showed the same morphological heterogeneity of the parental cells, but differed from one another in the degree of differentiation.

Multinucleated myotube-like structures were strongly stained by anti-desmin antibody; most mononuclear cells were weakly stained. About 80% of RMZ and cloned cells were scored as desmin-positive in cytocentrifuged preparations. The expression of embryonic myosin heavy chain, specifically recognized by the monoclonal antibody BF-G6, was found in RMZ cell line and was localised in the myotube-like structures. Only a few giant cells and rare mononucleated polygonal cells were stained. The average proportion of BF-G6 positive cells in cytocentrifuged preparations was of about 6% of the total RMZ cells. In the two RMZ clones studied, the expression of embryonic myosin was correlated to the proportion of myotube-like structures: a BF-G6 positivity of 35% was found in the most differentiated one.

Rhabdomyosarcoma is the most common soft tissue sarcoma in childhood and represents between 5% and 15% of all malignant solid tumours in children under 15 years of age. Rhabdomyosarcomas are subdidived into alveolar, embryonal and pleomorphic types (Enzinger & Weiss, 1983).

Only a few human rhabdomyosarcoma cell lines have been recently studied (McAllister *et al.*, 1969; Giard *et al.*, 1973; Chapman *et al.*, 1974; McAllister *et al.*, 1975); whenever histologic type is specified, the original tumour was an embryonal rhabdomyosarcoma. Moreover, *in vitro* differentiation properties of human rhabdomyosarcoma cells have not yet been fully characterized. Therefore, a rhabdomyosarcoma cell line of alveolar origin with a residual ability to differentiate *in vitro* could be a useful tool for elucidating the differentiation process in neoplastic and normal muscle.

The cell line described here, RMZ, was derived from a bone marrow metastasis of a human alveolar rhabdomyosarcoma. In this paper we present data on its *in vitro* growth, unique differentiation properties and the expression of embryonic myosin in the parental cells and in clonal derivatives obtained from the second *in vitro* passage.

Correspondence: P. Nanni.
Received 7 May 1986; and in revised form 25 June 1986.

### Materials and methods

### *Establishment of the RMZ cell line*

The culture was established from a biopsy of a bone marrow metastasis. The primary tumour, diagnosed as an alveolar rhabdomyosarcoma (Istituto di Anatomia e Istologia Patologica, Università di Padova, Italy), developed in the left thigh of a 2-year-old caucasian male child and was removed one year prior to metastasis. The patient was treated with chemo- and radiotherapy.

### *Culture procedures*

Cells were routinely cultured in Dulbecco's modified Eagle medium (GIBCO, Paisley, Scotland) supplemented with 100 U ml$^{-1}$ penicillin, 100 $\mu$g ml$^{-1}$ streptomycin (hereafter referred to as DMEM) and with 10% foetal calf serum (FCS, GIBCO). Horse serum (GIBCO) was used instead of FCS for the early *in vitro* passages and to study

© The Macmillan Press Ltd., 1986

*in vitro* differentiation (Blau & Webster, 1981). Cell cultures were maintained at 37°C in a humidified 5% $CO_2$ atmosphere. RMZ cells were monitored for mycoplasma contamination by fluorescent staining with Hoechst 33258 (Chen, 1977) and found to be mycoplasma-free.

### In vitro *growth properties*

The following parameters of *in vitro* growth were assessed as reported (Nanni *et al.*, 1983): cell doubling time in the logarithmic growth phase; saturation density (maximum cell number $cm^{-2}$); cloning efficiency (after seeding $2 \times 10^2$–$10^5$ cells in a 60 mm Petri dish) and mean cell diameter (determined on trypsinized cells with the aid of a micrometer).

### *Chromosome studies*

Exponentially growing cells were harvested with trypsin-EDTA and treated for 3 h with 0.33 $\mu g\,ml^{-1}$ colcemid (GIBCO). Cells were then pelleted, swollen for 15 min at 37°C with 75 mM KCl, and repeatedly fixed in cold methanol:acetic acid 3:1 for 15 min. The cells were then dropped onto glass slides and stained with a 3% Giemsa solution. The cytogenetic analysis was kindly performed by Dr. N. Testoni and Dr. A. Zaccaria (Istituto di Ematologia L. & A. Seràgnoli, Università di Bologna).

### *Immunofluorescence and immunocytochemical staining*

Desmin was identified with the monoclonal antibody DE-B-5 (Boehringer Mannheim, W. Germany) and with a polyclonal antiserum (DAKO, Santa Barbara, CA, USA) with identical results. BF-G6 is a monoclonal antibody which reacts specifically with embryonic-type myosin heavy chain present in foetal but not in neonatal or adult human skeletal muscle (Schiaffino *et al.*, 1986).

Cell cultures and cytocentrifuged preparations were fixed with a 7:3 mixture of acetone:methanol and processed for the indirect immunofluorescence assay, or for immunoperoxidase staining, using the avidin-biotin complex (Hsu *et al.*, 1981*a*, *b*).

Controls included non-immune sera and positive and negative tissues and the following cell lines: TG (Hernandez-Verdun *et al.*, 1984), a human epithelioid tumour cell line (kindly given by Dr. A. Pession, Istituto di Patologia Generale, Università di Bologna, Italy); EUE (De Carli & Larizza, 1978), a human epithelioid tumour line (kindly provided by Dr. P. Rocchi, Istituto di Cancerologia, Università di Bologna, Italy); Balb/3T3 clone A31, a cell line obtained from normal mouse embryonal fibroblasts (purchased from The American Type Culture Collection, Rockville, MD, USA).

### Results

### In vitro *growth properties*

The original tumour from which RMZ cells were derived was diagnosed as an alveolar rhabdomyosarcoma by current accepted criteria (Enzinger & Weiss, 1983). The tumour consisted of large sheets of cells separated by fibro-vascular septa. The tumour cells in the centre were frequently necrotic. The neoplastic population was formed by two different cell types: one constituted by round small elements having scanty cytoplasm; other cells, which were in the minority, displayed large eosinophilic cytoplasm and occasionally appeared multinucleated.

RMZ cells were adapted to grow in DMEM supplemented with 10% FCS and were routinely subcultured approximately once a week at dilutions from 1:2 to 1:4. *In vitro* growth characteristics were studied around the 20th passage, in order to study a population as close as possible to the original tumour. Basic features remain unchanged during subsequent culture passages (RMZ cells are now around the 40th *in vitro* passage).

Adherent cell growth was found to be dependent on seeding density: when the seeding concentration was between 8 and 4,000 cells $cm^{-2}$, a cloning efficiency $\leq$0.01% was observed, whereas at higher seeding density mass growth was observed. RMZ cells, seeded at 20,000 cells $cm^{-2}$, had a doubling time of $\sim$48 h and a saturation density of $1.6 \times 10^6$ cells $cm^{-2}$; mean cell diameter, measured in subconfluent cultures, was $15 \pm 0.24\,\mu m$ (range 9 to 21).

Chromosome analysis, performed on 44 metaphases, revealed a modal chromosome number of 84 (range 72 to 99). This hypotetraploid pattern is in agreement with that of another case of human alveolar rhabdomyosarcoma (studied on freshly-collected material) reported in the literature (Seidal *et al.*, 1982). Forty metaphases carried structural aberrations: dicentrics, acentric fragments, double minutes, chromosome and chromatid breaks. Sixteen metaphases were photographed and karyotyped: 6 of them were characterized by abnormalities of chromosome #3 but with different breakpoints; 6 metaphases carried abnormalities of chromosome #1 (5 of 1q). Moreover, in 2 metaphases a double 12q+ was observed. Additional abnormalities involved chromosomes #5, #14 and #17. Several markers of different shape were observed in most metaphases. (De Giovanni *et al.*, submitted for publication).

Attempts to induce tumours in nude mice with a subcutaneous injection of RMZ cells have insofar been unsuccessful.

### In vitro *morphology and differentiation properties*

Different cell types were present in RMZ cultures: small mononucleated polygonal or spindle-shaped, actively proliferating, cells were observed along with a few multinucleated elongated cells, resembling myotube structures, and large giant, sometimes multinucleated, elements (Figure 1). Myotube-like structures were usually not present in freshly seeded culture and became more evident during the late logarithmic phase and in confluent cultures. They usually appeared to contain $\geq 4$ nuclei; however, a precise estimate of the average number of nuclei was hardly possible in cultures because of the extensive criss-cross growth pattern of these cells. The capacity of mononucleated elements to give rise to myotube-like structures was constantly observed during *in vitro* passages of RMZ cells; the expression of this characteristic was found to be dependent on seeding density and serum concentration. The lower the values these two variables had, the stronger was the tendency of cultured cells to become elongated and fuse. The growth pattern of RMZ cells seeded at a concentration of 6,000 cells $cm^{-2}$ and cultured for 1 month is shown in Figure 2. Almost all the cells tended to a fusiform shape and to a mitotic arrest. RMZ cells, when cultured in the presence of 2% horse serum, revealed a pronounced increase in fusiform morphology.

We tried to isolate clones (Nanni *et al.*, 1983) from RMZ colonies grown in semisolid medium (either agar or agarose 0.33%), but we did not obtain any appreciable cell growth on plastic. This could be due to the small dimension of the agar colonies (McAllister *et al.*, 1975).

**Figure 1** RMZ cells in continuous culture (passage 25). Arrows: a, myotube-like structure; b, giant cell. Phase contrast, 100×.

**Figure 2** Low-density culture of RMZ cells. Micrograph was taken 30 days after seeding 6,000 cells $cm^{-2}$ in DMEM additioned with 10% FCS. Phase contrast, 100×.

From a low density seeding (400 cells $cm^{-2}$) of the second *in vitro* subculture of RMZ cells, a few colonies were obtained which were separately collected with a rubber policeman and reseeded as above to obtain RMZ clonal derivatives.

An interesting feature of the set of clones derived from RMZ is that they showed, under identical culture conditions, different overall morphology, *in vitro* growth rate and degree of myotube formation. Two extreme clones are shown in Figure 3: clone RC2 had a low differentiative trend, being composed almost exclusively of small polygonal cells, and a relatively high proliferative rate (with a doubling time of ~38 h), whereas clone RC5 showed a high degree of myotube formation and a very low proliferative rate (doubling time >100 h), which made its *in vitro* propagation very difficult.

Even though clones showed peculiar differentiative trends, they showed the occurrence of all the cell types described in the parental RMZ line (in quite different proportions). This suggests that the morphological heterogeneity described above is mainly due to the peculiar ability to differentiate *in vitro* and not to pre-existing, stable morphological variants, as observed in other experimental tumours (Dexter *et al.*, 1978; Lollini *et al.*, 1984).

### *Markers of myogenic differentiation*

In order to better define the *in vitro* differentiative properties of rhabdomyosarcoma cells, we studied the expression of desmin and embryonic myosin in RMZ and cloned cells by means of immunofluorescence and immunoperoxidase techniques.

**Figure 3** Clonal derivatives RC2 (a) and RC5 (b) obtained from the second *in vitro* passage of RMZ cells. Both clones were cultured in DMEM additioned with 10% FCS. Phase contrast, 100 ×.

In the RMZ cell line the anti-desmin antibody strongly stained almost all the myotube-like structures (Figure 4); the brightest fluorescence labelling was seen at the ends of myotube-like structures. Small (polygonal and spindle-shaped) cells showed a gradation of staining from completely negative to strongly positive elements. Giant multinuclear elements were sometimes desmin-positive; fluorescent filaments were particularly evident in these cells. In order to obtain a precise estimate of the proportion of RMZ cells expressing desmin, cytocentrifuged preparations were stained by immunofluorescence. The proportion of desmin-positive cells in cytocentrifuged preparations was 81.3% ± 2.9 (mean ± s.e. of 4 independent experiments). Control experiments were performed with cell lines of human (TG and EUE) and murine origin (3T3), all of which were completely negative.

**Figure 4** RMZ cells stained with anti-desmin antiserum. Immunofluorescence, 500 ×.

The RMZ cell line has been characterised for the expression of embryonic myosin, as shown by BF-G6 reactivity. All the myotube-like structures showed a strong positivity (Figure 5a) with fluorescent filaments and sometimes cross-striations (Figure 5b). Almost all the small cells were negative; giant cells were sometimes positive. In standard culture conditions the proportion of myosin-positive cells in cytocentrifuged preparations was 6.3% ± 0.9 (mean ± s.e. of 3 experiments). Control cell lines (TG, EUE, 3T3) were completely negative.

The expression of embryonic myosin heavy chain was investigated also in the two RMZ clonal derivatives previously described (see Figure 3), studied under standard culture conditions. Clone RC2 showed a BF-G6 positivity (Table I) not far from that of the uncloned parental cell line, whereas a strongly higher proportion of embryonic myosin-expressing cells was found in clone RC5. Therefore, as previously reported for cell morphology, the study of RMZ clones also showed that the expression of embryonic myosin was not clonally distributed, but was rather correlated to the proportions of myotube-like structures of the culture.

**Figure 5** RMZ cells stained with monoclonal anti-embryonic myosin heavy chain antibody BF-G6: (a) immunoperoxidase, 50 ×; (b) cross striations, immunofluorescence, 1,250 ×.

Both RMZ clones studied (RC2 and RC5) showed high and comparable proportions of desmin-expressing cells in cytocentrifuged preparations (Table I).

### Discussion

The most interesting features of the new human cell line, RMZ, described here are the following: (1) it was derived from a rhabdomyosarcoma of the alveolar histological type; (2) it retained some ability to differentiate *in vitro*; and (3) *in vitro* morphological differentiation into myotube-like structures correlates with the expression of embryonic myosin heavy chain.

In the RMZ cell line some cells express a myosin heavy chain type antigenically similar to that present in human muscle during the early stage of development. RMZ cells are (at least partially) able to differentiate *in vitro* to long multinucleated myotube-like structures expressing both desmin and embryonic myosin. However, myosin- or desmin-negative cells were also represented in RMZ cell line. Further studies on the proliferative and differentiative ability and on the origin of the different cell types present in rhabdomyosarcoma might contribute to the understanding of the differentiation process in neoplastic and normal muscle. We are presently considering the distribution of desmin, embryonic myosin and other myosin isoforms and the use of modulating agents.

In RMZ cultures we observed a small fraction of large, flat, distended, sometimes multinucleated, cells which frequently exhibited immunoreactivity for desmin and embryonic myosin. It should be noted that cells morphologically similar to those described here have been observed in cell cultures

**Table I** Expression of embryonic myosin heavy chain and desmin in RMZ cell line and in its clonal derivatives

| | *Percentage of cytocentrifuged cells expressing*[a] | |
|---|---|---|
| *Cells* | *Embryonic myosin* | *Desmin* |
| RMZ cell line | 6.3±0.9 | 81.3±2.9 |
| Clone RC2 | 4.0 | 71.7 |
| Clone RC5 | 35.4 | 86.9 |

[a]By immunofluorescence.

obtained from Duchenne muscular dystrophy patients (Blau *et al.*, 1983) and in two other rhabdomyosarcoma cell lines (McAllister *et al.*, 1969; Chapman *et al.*, 1974). It is possible that the presence of such cells could be related with developmental alterations.

We obtained a set of clonal derivatives from the second *in vitro* passage of RMZ cells. These clones share with the parental cells all the features outlined above, but differ quantitatively from one another in the capacity to give rise to differentiated myotube-like structures. We are presently trying to study the reciprocal effects on differentiation of culture media conditioned by the various clones and of cell co-cultures.

In the RMZ cell line, proliferative and differentiative abilities seem to be opposite. In this model the study of inducers of differentiation and of stimuli causing quiescent, differentiated cells to re-enter the cell cycle could have therapeutic implications.

It is considered that a comparison of the *in vitro* properties of RMZ with those of other rhabdomyosarcoma cell lines, derived from embryonal tumours, could be useful for the study of correlations between the behaviour of *in vitro* cultured cells and the histologic classification of myogenic tumours.

*Note added in proof*

Progressive tumour growth was obtained in Swiss nude mice after a s.c. or i.m. injection of $3 \times 10^7$ cells of clone RC2.

The authors wish to thank Dr. N. Testoni and Dr. A. Zaccaria (Istituto di Ematologia L. & A. Seràgnoli, Università di Bologna) for performing cytogenetic analysis. G. Nicoletti is in receipt of a training grant from CNR, Roma, Italy. B. Del Re and C. Ceccarelli are in receipt of a fellowship from Associazione Italiana per la Ricerca sul Cancro, Milano, Italy. P.-L. Lollini is in receipt of a Ph.D. studentship (Dottorato di Ricerca in Oncologia) from Ministero della Pubblica Istruzione, Italy.

This work has been supported by grants from Ministero della Pubblica Istruzione, Italy, from Associazione Italiana per la Ricerca sul Cancro, Milano, Italy and from the Italian National Research Council, Special Project MPR, SP. 3 (contract number 8302823.56).

### References

BLAU, H.M. & WEBSTER, C. (1981). Isolation and characterization of human muscle cells. *Proc. Natl Acad. Sci. USA*, **78**, 5623.

BLAU, H.M., WEBSTER, C. & PAVLATH, G.K. (1983). Defective myoblasts identified in Duchenne muscular dystrophy. *Proc. Natl Acad. Sci. USA.*, **80**, 4856.

CHAPMAN, A.L., BOGNER, P. & BEHBEHANI, A.M. (1974). A study of a new human tumor cell line (rhabdomyosarcoma). *Proc. Soc. Exp. Biol. Med.*, **146**, 1087.

CHEN, T.R. (1977). *In situ* detection of mycoplasma contamination in cell cultures by fluorescent Hoechst 33258 stain. *Exp. Cell Res.*, **104**, 255.

DE CARLI, L. & LARIZZA, L. (1978). Induced chromosome variation in cultured cell populations. In *Origin and natural history of cell lines*, Barigozzi, C. (ed), p. 93, Alan R. Liss, Inc.: New York.

DEXTER, D.L., KOWALSKI, H.N., BLAZAR, B.A., FLIGIEL, Z., VOGEL, R. & HEPPNER G. (1978). Heterogeneity of tumor cells from a single mouse mammary tumor. *Cancer Res.*, **38**, 3174.

ENZINGER, F.M. & WEISS, S.W. (1983). *Soft Tissue tumours*. The C.V. Mosby Co., St. Louis.

GIARD, D.J., AARONSON, S.A., TODARO, G.J. & 4 others (1973). *In vitro* cultivation of human tumors: establishment of cell lines derived from a series of solid tumors. *J. Natl Cancer Inst.*, **51**, 1417.

HERNANDEZ-VERDUN, D., DERENZINI, M. & BOUTEILLE, M. (1984). Relationship between the Ag-NOR Proteins and ribosomal chromatin *in situ* during drug-induced RNA synthesis inhibition. *J. Ultrastruct. Res.*, **88**, 55.

HSU, S.M., RAINE, L. & SANGER, H. (1981a). A comparative study of the PAP method and avidin-biotin-complex method for studying polypeptide hormones with radioimmunoassay antibodies. *Am. J. Clin. Pathol.*, **75**, 734.

HSU, S.M., RAINE, L. & SANGER, H. (1981b). The use of avidin-biotin-peroxidase complex (ABC) in immunoperoxidase techniques: a comparison between ABC and unlabelled antibody (PAP) procedures. *J. Histochem. Cytochem.*, **29**, 577.

LOLLINI, P.-L., DE GIOVANNI, C., EUSEBI, V., NICOLETTI, G., PRODI, G. & NANNI, P. (1984). High-metastatic clones selected *in vitro* from a recent spontaneous BALB/c mammary adenocarcinoma cell line. *Clin. Expl. Metastasis*, **2**, 251.

McALLISTER, R.M., MELNYK, J., FINKLESTEIN, J.Z., ADAMS, E.C. Jr. & GARDNER, M.B. (1969). Cultivation *in vitro* of cells derived from a human rhabdomyosarcoma. *Cancer (Phila.)*, **24**, 520.

McALLISTER, R.M., NELSON-REES, W.A., PEER, M. & 5 others (1975). Childhood sarcomas and lymphomas. *Cancer*, **36**, 1804.

NANNI, P., DE GIOVANNI, C., LOLLINI, P.-L., NICOLETTI, G. & PRODI, G. (1983). TS/A: A new metastasizing cell line from a BALB/c spontaneous mammary adenocarcinoma. *Clin. Expl. Metastasis*, **1**, 373.

SCHIAFFINO, S., GORZA, L., SARTORE, S., SAGGIN, L. & CARLI, M. (1986). Embryonic myosin heavy chain as a differentiation marker of developing human skeletal muscle and rhabdomyosarcoma. A monoclonal antibody study. *Exp. Cell Res.*, **163**, 211.

SEIDAL, T., MARK, J., HAGMAR, B. & ANGERVALL, L. (1982). Alveolar rhabdomyosarcoma: a cytogenetic and correlated cytological and histological study. *Acta Path. Microbiol. Immunol. Scand. Sect. A*, **90**, 345.